# Cross-sectional survey of older peoples' views related to influenza vaccine uptake

**DOI:** 10.1186/1471-2458-6-249

**Published:** 2006-10-11

**Authors:** Punam Mangtani, Elizabeth Breeze, Sue Stirling, Smita Hanciles, Sari Kovats, Astrid Fletcher

**Affiliations:** 1 Department of Epidemiology and Public Health, London School of Hygiene and Tropical Medicine, Keppel Street, London, WC1E 7HT, UK; 2 Department of Epidemiology and Public Health, University College London 1-19 Torrington Place, London WC1E 6BT, UK; 3 Department of Public Health and Policy, London School of Hygiene and Tropical Medicine, Keppel Street, London, WC1E 7HT, UK

## Abstract

**Background:**

The population's views concerning influenza vaccine are important in maintaining high uptake of a vaccine that is required yearly to be effective. Little is also known about the views of the more vulnerable older population over the age of 74 years.

**Methods:**

A cross-sectional survey of community dwelling people aged 75 years and over wh, previous participant was conducted using a postal questionnaire. Responses were analysed by vaccine uptake records and by socio-demographic and medical factors.

**Results:**

85% of men and 75% of women were vaccinated against influenza in the previous year. Over 80% reported being influenced by a recommendation by a health care worker. The most common reason reported for non uptake was good health (44%), or illness considered to be due to the vaccine (25%). An exploration of the crude associations with socio-economic status suggested there may be some differences in the population with these two main reasons. 81% of people reporting good health lived in owner occupied housing with central heating vs. 63% who did not state this as a reason (p = 0.04), whereas people reporting ill health due to the vaccine was associated with poorer social circumstances. 11% lived in the least deprived neighbourhood compared to 36% who did not state this as a reason (p = 0.05) and were less likely to be currently married than those who did not state this as a reason (25% vs 48% p = 0.05).

**Conclusion:**

Vaccine uptake was high, but non uptake was still noted in 1 in 4 women and 1 in 7 men aged over 74 years. Around 70% reported they would not have the vaccine in the following year. The divergent reasons for non-uptake, and the positive influence from a health care worker, suggests further uptake will require education and encouragement from a health care worker tailored towards the different views for not having influenza vaccination. Non-uptake of influenza vaccine because people viewed themselves as in good health may explain the modest socio-economic differentials in influenza vaccine uptake in elderly people noted elsewhere. Reporting of ill-health due to the vaccine may be associated with a different, poorer background.

## Background

Influenza is a significant cause of winter mortality as well as acute incapacity in elderly people. Complications are unpredictable but more common in older adults, who may need urgent hospitalisation or substantial increases in home care. The epidemic of 1989 was estimated to result in about 25,000 additional deaths in England and Wales, 80% of whom were over the age of 74[1]. Vaccination is currently the most effective measure against influenza. The use of killed influenza vaccine significantly reduces morbidity and mortality in elderly people [2-5].

Since 1998 all over 74 year olds in the UK have been offered influenza vaccination by their general practitioners (GPs). This was then extended to all over 64 year olds in 2000. Influenza vaccine uptake in all over 65 year olds is monitored with an uptake of 60% achieved in 2000, and over 70% in subsequent years[6]. Although a number of previous surveys have suggested non-receipt of vaccination is linked to a perception the vaccine would make them ill [7-12] others have suggested non-receipt of vaccine uptake in over 64 year olds was also lower in those who judged their own health to be good[13]. Information on the prevalence of such a view towards influenza vaccine in the UK in the more vulnerable population aged over 74 years is not known. Such information would help focus efforts on further improving and maintaining high uptake in this older age group.

We conducted a cross-sectional postal survey in community based over 74 year olds in 2001 soon after eligibility for influenza vaccine was extended to age 65. We examined the associations between self perceived health, activity and influenza vaccine uptake. We also explored associations between the main reasons for refusing vaccine and socio-economic background, an aspect rarely covered in the literature.

## Methods

The subjects were patients aged over 74 years from practices in the Medical Research Council Trial of Assessment and Management of Older People in the Community [14] and who were also taking part in a sub study of influenza vaccination[15]. In brief the trial was conducted in general practice with practices randomised to providing either universal assessment or targeted assessment of people aged 75[16]. All participants received a brief multidimensional assessment followed by a nurse-led in-depth assessment in the universal group, whereas in the targeted group the in-depth assessment was offered only to those with problems established at the brief assessment. The in-depth medical assessment included questions on underlying medical conditions and the following socio-economic information: housing tenure, area of residence based on the Carstairs deprivation Index[17], population density of area, smoking status, and marital status. Only participants in practices in the universal arm of the trial were included (n = 53 practices) as this arm represented an unselected sample of all older people aged 75 and over on the practice lists (apart from people resident in nursing homes or long stay hospital care who were excluded). The response rate of individuals to the assessment in the universal arm was 78%. 34 of the 53 practices in the universal arm also took part in the flu vaccine study, providing data on influenza vaccine uptake at the individual level. For the survey, a two stage self-weighted sample (sampling first the practices, probability proportional to size, and then the individuals in the practices) was taken such that each patient had an equal chance of being sampled. Practice lists updated in 2001 were used and people who had died or moved away were excluded. Information on influenza vaccine receipt was obtained from GP records. The questionnaires were sent from the practice with a letter signed by the patient's GP after excluding any patients who were too ill to be approached. Replies were returned to the research team at LSHTM using prepaid addressed envelopes. It was emphasised that any replies were confidential and would not be seen by health personnel at the practice.

**Figure 1:**
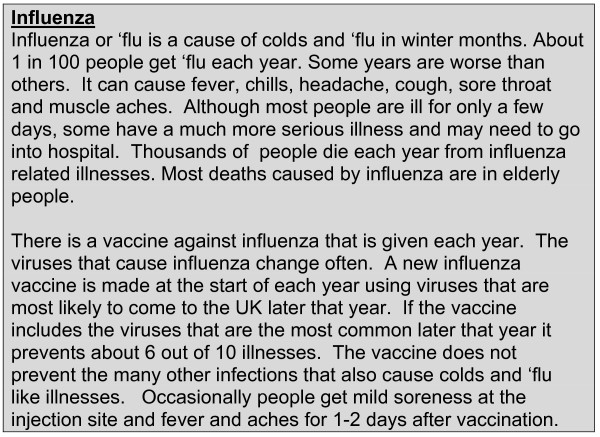
Patient information provided on influenza and influenza vaccination.

The short questionnaire asked respondents whether they had had influenza vaccination last year. Reasons for having or not having influenza vaccination were ascertained using a list of closed-ended questions together with an open-ended option. A description of the illness, the vaccine and its effectiveness, was then given (figure 1) followed by a question on future vaccination intentions. They were also asked to assess their level of general health and activity compared to other people of their own age. A second questionnaire was sent to non-responders after two weeks. Ethics approval for the survey was obtained from the then Multi-Centre Research Ethics Committee (MREC). Return of the completed questionnaire signified consent.

Influenza vaccine status confirmed by GP records was used to examine patients' main reasons for having or not having influenza vaccination. The most common reasons were examined further in relation to information on underlying health and socio-economic status collected in the main trial. All analyses were conducted taking into account clustering of subjects at the practice level (i.e. the possible lack of independence of observations in subjects which may be more correlated than if individuals were selected at random) by using the svy suit of commands in Stata 7[18].

The sample size was based on a small survey of elderly people in 1999 by one of the authors (PM), where 40% did not have influenza vaccination[19]. The most common reason cited by those who did not have influenza vaccination (30% or 12% of the whole sample) was that they did not see themselves as at risk. A sample size of about 1,270 was aimed for. Based on simple random sampling 111 subjects would have been required to estimate a 12% prevalence of the perception that they were not at risk with 6% precision (i.e. a range of 6–18%) with 95% confidence. This was inflated by four to take into account clustering at the practice level [20] and increased to take into account an anticipated response rate of 70%, as seen in the earlier study. The numbers were then doubled to allow examination of subgroups, eg in practices that actively recalled patients for influenza vaccination, if necessary.

## Results

Of 1573 patients initially sampled, practice nurses noted that 67 had died, 121 had moved, and 142 were too ill to be contacted. Responses to the mailed questionnaire identified a further 10 who had since died, 24 who had moved or had wrong addresses and 3 who were too ill to reply. This left a total of 1206 eligible patients of whom 113 refused and 174 did not reply after a second mailing. Information on vaccine status in 2000 was missing in another 35 patients and a further 40 had not completed the prior full assessment. The effective response rate was therefore 75% (844/(1206-35-40)).

Of the 844 responders, 85% of 329 men and 75% of 515 women were vaccinated against influenza in 2000 according to GP records. This percentage was higher than in the whole sample (responders and non-responders) where it was 69.8% and 60.5% in men and women respectively. However, the age and sex patterns of the responders' recorded uptake were similar to that of the full sample[15]. Vaccine recipients were more likely to be male than female (RR 1.12, 95% CI 1.03–1.22) with a non-significant tendency for those aged 80–84 to be more likely to be vaccinated that those below the age of 80 years (1.04, 95% CI 0.9–1.2).

Over 93% of those who stated that they had had influenza vaccination in the last 12 months also had a GP record of this. Of those who had a GP record of vaccination last year, 92% recalled that they had been vaccinated, 4% thought it was more than a year ago, 2% were not sure and 1% thought the last time they had had influenza vaccine was a few years ago.

Recall of an invitation or being in a practice that actively recruited patients (latter information obtained from the practices) was high for both vaccinated and unvaccinated patients but was not significantly associated with vaccination in over 74 year olds in this sample. There was also no significant association with self-perceived health or inactivity (Table 1).

### Reasons given for influenza vaccine receipt

Most vaccinated patients indicated that a reason for being vaccinated was a recommendation from a health care worker (Table 2). Recommendation by a health care worker was defined to include opportunistic offers, face to face, letter or telephone contacts. A much smaller percentage reported recommendation from a friend or relative. Nearly all those who had had influenza vaccination agreed either with the statement that prevention was better than cure or that they were aware of the benefits, a quarter perceived themselves at high risk and an even smaller percentage noted that they had had a bad experience with influenza in the past

### Reasons given for influenza vaccine non-receipt

Of the pre-coded options given the most popular reason for non receipt of vaccine was that they did not get ill, followed by the perception that the vaccine makes them ill or they lacked interest in influenza vaccination (Table 2). Fewer than one in ten did not like injections or thought the vaccine did not work. Perceived allergies to vaccination were not important.

Of those who had not had the vaccine last year, a substantial percentage reported that they had never had the vaccine (30%). A small percentage appeared to lack knowledge that they needed it each year (6.5%) or did not know they could have the vaccine (5.3%). A few also volunteered that they thought they were too old for the vaccine (5.3%). Information given about influenza, the vaccine and how effective it is (figure 1) was noted to be useful in 80% (95%CI 76–84%) of those who had had the vaccine last year and 45% (35–55%) who had not. Of those who had not had the vaccine last year, 29% (95% CI 20–40%) said they would have it next season. Of these, 96% said the information was useful compared to 27% of those who would not have the vaccine next year. Of the 71% who said they would not have the vaccine next year, the order of importance of attitudes associated with not having vaccination next year was similar to the whole group of not vaccinated.

In this sample few reported physical barriers such as lack of transport, and only one person gave reasons of unsuitable surgery times or that the surgery was too far away.

### Characteristics of people with particular reasons against vaccination

The crude association between the two main reasons against influenza vaccination and socio-economic status as well as underlying health were explored (table 3). A statement that they were generally healthy was associated with living in owner occupied housing with central heating, not living in urban areas and not having a history of past influenza. There was also a non significant association with being non-smokers. People who reported that they did not have the vaccine because it might make them ill were less likely to be currently married, and less likely to live in comfortable neighbourhoods.

## Discussion

Influenza vaccine uptake in 75 year olds and older did not appear to be related to perception of their health or activity levels. Of those who had not had the vaccine 70% reported they would not have the vaccine the following year. The commonest reasons for not having had influenza vaccination in this population of over 74 year olds was that they judged their own health to be good. A smaller percentage thought the vaccine could make them ill.

The differences in some socio-demographic factors associated with these two reasons for not having influenza vaccination suggest that the perceptions are distinct in the way they evolve. It appears that people who felt they did not need influenza vaccine because they had excellent health were likely to be better supported in terms of marital status and socio-economic status. In contrast peoples' perceptions that the vaccine makes them ill were associated with poorer social circumstances. The lack of uptake of influenza vaccine for differing reasons by people with different material backgrounds may explain why the socio-economic differentials in influenza vaccine uptake noted elsewhere are modest[15].

In the survey, self report of influenza vaccine was confirmed in patient medical records in the great majority. Over 93% of peoples' recalls of influenza vaccination were verified by health centre records confirming that influenza vaccination, as reported by patients, is highly reliable. The same level of reliability was seen in a study of over 64 year olds in Finland[9] and in a different study of over 64 year olds in the UK[21].

The attitudes to past vaccination seen here were related similarly to future intentions re influenza vaccination. Although we were not able to test their predictive value, others have noted a clear relationship between intention and actual uptake of influenza vaccine[22].

The respondents to the postal survey were people who may be less typical than the general population of over 74 year olds because they were willing to take part in this survey. They were also more likely to be vaccinated than those who did not complete a questionnaire so that the prevalence of negative attitudes to influenza vaccination is likely to be underestimated compared to the prevalence in the general population. It is also possible that the association between non-receipt of vaccination and less favourable socioeconomic circumstances is under-estimated.

The high percentage who accepted uptake associated with a recommendation by a health care worker is consistent with other studies [9,11,23] especially among those who have not had previous vaccination [24].

Studies in other settings have also shown that thinking that the vaccine makes them ill was associated with non-receipt of vaccination [7-12], Our findings in over 74 year olds mirrors that of the Dutch study of over 64 year olds which showed that receipt of vaccine uptake was lower in those who judged their own health to be good[13] Our findings are also consistent with results from qualitative interviews of people over 74 years of age in the UK. The decision to be vaccinated against influenza was thought to be based on beliefs about whether it would prevent colds and influenza or cause colds and influenza and not on whether they saw themselves as at high risk. Of interest is that those with positive views of influenza vaccine saw any post vaccine ill health either as coincidence or were happy to continue with professional advice[25].

In Sweden and the US influenza vaccine uptake is higher in those with higher levels of education[26,27], however, a Canadian telephone survey did not find uptake to be significantly related to education[8]. They also saw no difference in uptake according to household income. This difference in findings maybe because of a greater effort by health care workers to promote vaccination in Canada. We were not able to look at education levels. We instead explored the links between perceptions of influenza vaccine and social and health related backgrounds and suggest that, underlying the documented socio-economic differentials in influenza vaccine uptake [15,28], there are divergent attitudes.

A belief that the vaccine can make one ill appears be associated with socio-demographic patterns consistent with reduced access or ability to act upon accurate knowledge. People who think they do not need the vaccine because they are generally healthy appear to have good social support in terms of being currently married and there is some indication of more healthy behaviours. Education at older ages would be reasonably expected to reduce both beliefs that the vaccine lacks effect or that it gives rise to illness.

## Conclusion

Health workers have a positive influence on influenza vaccine uptake and should continue to try to advise people and give individual education to all who refuse influenza vaccine, not just those who think the vaccine might make them ill. An important message for all those who decline influenza vaccine is the ability for even seasonal influenza to change from year to one where there maybe little protective immunity in the population

This work also provides an initial insight into the possible backgrounds in which particular perceptions of influenza vaccine might occur and needs to be confirmed elsewhere. Changes in the prevalence of such perceptions and associations with socio-economic status as a result of increasing experience of the health service offering vaccination against seasonal influenza each year as well as worries over an influenza pandemic would also be of interest.

## Competing interests

The author(s) declare that they have no competing interests.

## Authors' contributions

PM with EB and AF conceived, designed the study and interpreted the findings. PM took the lead in writing the paper. SS advised on the practice populations, sampling methods and practice recruitment. EB and SS provided advice on the analysis plan. SH helped design and pilot the questionnaire and was responsible for the conduct of the cross-sectional study. SK collected and analysed the vaccine coverage data. All authors read and approved the final manuscript.

## Pre-publication history

The pre-publication history for this paper can be accessed here:



## Figures and Tables

**Table 1:** Characteristics of patients by vaccination status

**Characteristic**	**Not vaccinated **n = 169	**Vaccinated **N = 675	**RR adjusted for age, sex**	**95% CI**	**p value**
	Number (%)	Number (%)			
Practice actively recruits patients for influenza vaccination	135 (80)	537 (79.6)	1.00	0.88, 1.14	0.98
Patient recalls receiving letter/phone call to have vaccine	80 (47)	359 (53)	0.95	0.88, 1.04	0.26
					
**Perception of own health**					
Excellent	17 (10.3)	48 (7.1)	1		
Very good	43 (26.1)	184 (27.4)	1.11	0.95, 1.29	
Good	55 (33.3)	231 (34.4)	1.10	0.95, 1.28	
Fair	34 (20.6)	174 (25.9)	1.15	0.94, 1.40	
Poor	16 (9.7)	34 (5.1)	0.94	0.72, 1.21	0.16
					
**Perception of own activity levels**					
Very active	27 (16.3)	103 (15.4)	1		
Active	76 (45.8)	334 (49.8)	1.03	0.97, 1.16	
Not very active	34 (20.5)	175 (26.1)	1.07	0.93, 1.22	
Not active	29 (17.5)	58 (8.7)	0.87	0.67, 1.12	0.07

**Table 2:** Patients' reasons for having or not having influenza vaccination

	**N**	**%**	**95% CI**	
**Reasons for having vaccine (N = 675)**				
Positive attitude to prevention^1^	588	87.1	84.4	89.8
Recommended by HCW	559	82.8	78.1	86.7
Recommended by friend/relative	73	10.8	9.2	12.7
Perceived themselves at higher risk because of an underlying medical condition	166	24.6	21.7	27.9
Perceived bad past experience with influenza	120	17.8	15.2	20.8

**Reasons for not having vaccine (N = 169)**				

**Attitudes to vaccination**				
Don't get ill	75	44.4	37.4	51.9
Perception vaccine makes them ill	43	25.4	18.1	32.8
Not interested	28	16.6	11.6	22.3
Do not like injections	19	11.2	7.1	17.9
Perception would not work	21	12.3	8.1	19.3
Allergy to vaccine	9	5.3	3.3	8.8
Did not get around to it	9	5.3	3.0	9.5
				
**Knowledge**				
Never had it before	51	30.2	22.2	38.1
Did not know needed each year	11	6.5	3.3	10.8
Volunteered thought they were too old	9	5.3	2.1	8.4
Did not know could have it	5	5.3	1.2	7.2
				
**Physical barriers**				
No transport	11	6.5	3.8	11.3
Unable to attend because of ill health	9	5.3	2.6	11.1
Time of clinics not suitable	1	0.6	0.1	4.5
Too far away	1	0.6	0.1	4.5

^1^Agreed they were aware of benefits or prevention better than cure

**Table 3:** Characteristics of people with the two most common reasons for not having influenza vaccine

**Characteristic**	**Stated did not get ill vs rest** **(n = 50/107)**	**Stated might make them ill vs rest ** **(n = 28/107)**
Married	52 vs 33% p = 0.15	25 vs 48% p = 0.032
Urban residence	10 vs 25% p = 0.007	14 vs 19% p = 0.66
Never smoked	48 vs 35% p = 0.2	39 vs 42% p = 0.86
Least deprived neighbourhood	22 vs 30% p = 0.29	11 vs 32% p = 0.049
Owner occupied with central heating	81 vs 63% p = 0.037	71 vs 74% p = 0.65
Had experience of flu	38 vs 75% p = 0.0004	71 vs 53% p = 0.12
Respiratory illness	58 vs 46% p = 0.33	36 vs 57% p = 0.18
Cardiovascular illness	88 vs 81% p = 0.18	11 vs 18% p = 0.31
